# Monoamine content during the reproductive cycle of *Perna perna* depends on site of origin on the Atlantic Coast of Morocco

**DOI:** 10.1038/srep13715

**Published:** 2015-09-09

**Authors:** Mounia S. Klouche, Philippe De Deurwaerdère, Françoise Dellu-Hagedorn, Nouria Lakhdar-Ghazal, Soumaya Benomar

**Affiliations:** 1 Unit of Research on Biological Rhythms, Neuroscience and Environment; Faculty of Sciences, Mohammed V University, Rabat, Morocco; 2 Univ. de Bordeaux, Institut des Maladies Neurodégénératives UMR 5293, 33000 Bordeaux, France.; 3 CNRS, Institut des Maladies Neurodégénératives UMR 5293, 33000 Bordeaux, France.; 4 CNRS, Institut des Neurosciences Cognitives et Intégratives d’Aquitaine UMR 5287.

## Abstract

Bivalve molluscs such as *Perna perna* display temporal cycles of reproduction that result from the complex interplay between endogenous and exogenous signals. The monoamines serotonin, dopamine and noradrenaline represent possible endocrine and neuronal links between these signals allowing the molluscs to modulate reproductive functions in conjunction with environmental constraints. Here, we report a disruption of the reproductive cycle of mussels collected from two of three sites along the Moroccan atlantic coast soiled by industrial or domestic waste. Using high pressure liquid chromatography, we show that the temporal pattern of monoamine content in the gonads, pedal and cerebroid ganglia varied throughout the reproductive cycle (resting, developing, maturing, egg-laying) of mussels from the unpolluted site. Marked modification of monoamine tissue content was found between sites, notably in noradrenaline content of the gonads. Discriminant statistics revealed a specific impact of mussel location on the temporal variations of noradrenaline and serotonin levels in gonads and cerebroid ganglia. Correlation analyses showed profound and temporal changes in the monoamine content between organs and ganglia, at the two sites where the reproduction was disrupted. We suggest that environmental constraints lead to profound changes of monoaminergic systems, which thereby compromises the entry of mussels into their reproductive cycle.

Organisms such as bivalve molluscs display temporal cycles of reproduction, implying the existence of complex interactions of endogenous and exogenous factors. The suite of reproductive functions is necessary for species survival and is extremely demanding for these organisms. It requires multiple control processes, allowing the entry of animals into the sexual cycle from the onset of gametogenesis to gamete release. Monoamine concentrations in invertebrate tissues have been reported to display seasonality and could be involved in the reproductive cycle in several bivalves^1^,^2^. These monoamine systems could in turn integrate endogenous and exogenous factors and display high sensitivity with respect to environmental factors.

The role of monoamines during the reproductive cycle in bivalves is not well known^3^, in part because monoamine content differs between species^4^,^5^,^6^. In the Pacific oyster *Crassostrea gigas*^7^ and the scallop *A. purpuratus*^8^, the lowest concentrations of dopamine (DA) and serotonin (5-HT) were detected during the resting and maturing reproductive stages and the highest levels when the gonads are ripe. Concentrations were found to drop suddenly at spawning. Conversely, in the gonads of *Spisula solidisima*, 5-HT concentrations are low during gonad development and increase after spawning^9^. The endocrine function of gonads in marine bivalves is thought to be regulated through monoamine neurosecretions arising from the cerebroid ganglia, but not the pedal ganglia^6^,^10^,^11^,^12^. In addition to a specific role in reproduction, monoamines are also involved in numerous other functions. In all bivalve species, the reproductive cycle is associated with a dramatic increase in body mass and metabolic stress and, therefore, monoamines could be among the factors controlling organs.

Specific environmental factors are expected to alter the reproductive cycles of bivalves, with both ecological and economical implications. The reproduction of mussels like *Perna perna* is disrupted in some Moroccan sites, presumably by pollution, as revealed by a decrease in egg-laying events over the year^13^,^14^. Based on the putative role of monoamines throughout the reproductive cycle, we postulate that monoamine content and interaction are altered in mussels by specific environmental conditions.

In the present study, *Perna perna* mussels from 3 distinct sites on the Moroccan coast were collected and the monoamine content in the cerebroid ganglia, the pedal ganglia and the gonads was measured. Mussels were collected at four phases during the course of a year in correspondence with the different stages of the animal’s reproductive cycle. Levels of noradrenaline (NA), DA and 5-HT were measured using high pressure liquid chromatography coupled to electrochemical detection.

## Results

### The reproductive cycle of *Perna perna* at the three sites

**Figure 1:**
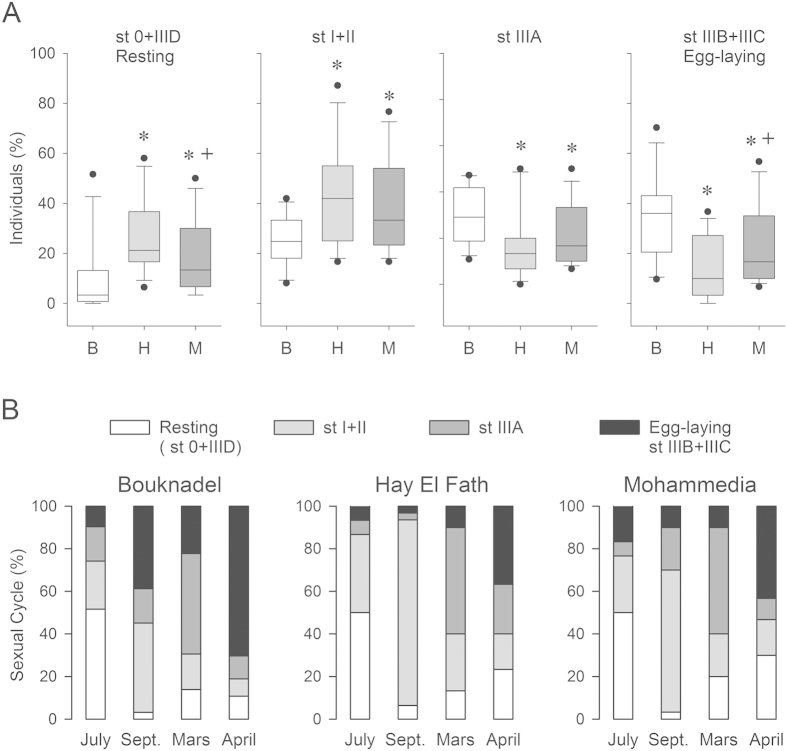
1A. Annual variations in sexual stages found in the gonads of *P. perna* after collection from the Mohammedia (M), Hay El Fath (H) and Bouknadel (B) sites. Classification of bivalve sexual stages according to Lubet (1959): StIIIC + IIIB, egg-laying; StIIIA, later development (maturation) + morphologically ripe; StI + II, early development; St0 + IIID), resting stage + mussel recently spent. The data correspond to the proportion of approximately 30 individuals collected each month in each site for a given sexual stage. The inter-site variability in the distribution for a sexual stage is illustrated by Tukey’s boxes. * and +p < 0.05 with respect to the distribution found at Bouknadel or Hay El Fath, respectively (Student Newman-Keuls). 1B. Distribution of the different stages in the sexual cycle for the months that were selected for the neurochemical study (July 2010, September (Sep.) 2010, March 2011 and April 2011 (see supplementary Fig. S1 for the full distribution over a year).

Figure 1 depicts the proportion of mussels in each of the four distinct states of the reproductive cycle at each site (Fig. 1A,B). Over an entire year (Fig. 1A, supplementary Fig. S1), mussels from the site at Bouknadel displayed a higher proportion of StIIIA (Kruskall-Wallis, H = 7.3, p < 0.05), StIIIB + IIIC (egg-laying, H = 10.6, p < 0.01) and a lower proportion of St0 + IIID (resting stage, H = 6.1, p < 0.05) or StI + II (H = 6.1, p < 0.05) compared with mussels from the other two sites (Fig. 1A). For the subsequent neurochemical study, we selected July, September, March and April as the most representative periods for each of the different sexual stages (Fig. 1B). The environmental conditions, including temperature, pH, salinity, conductivity of water and the quantity of heavy metals contained in samples of 50 mussels from each site is shown in supplementary Fig. S2 and S3. During a 13 month monitoring period, we found significantly lower values for the salinity (Kruskal-Wallis, H = 18.8, p < 0.001) and conductivity (H = 13.6, p = 0.001) at the Mohammedia site. Moreover, exposure of mussels to heavy metals differed at the three sites over a year, with higher concentrations of lead found at Mohammedia and Hay El Fath.

### Seasonal fluctuations of monoamines in *Perna perna*

**Figure 2:**
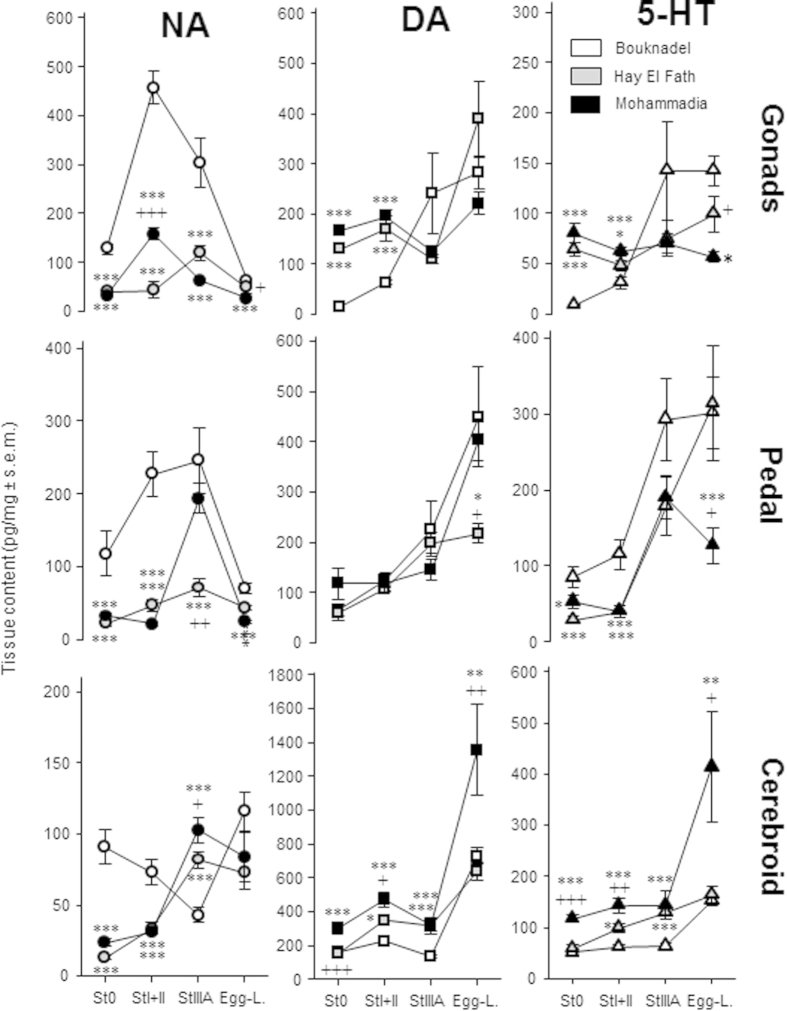
Distribution of monoamines noradrenaline (NA), dopamine (DA) and serotonin (5-HT) at the different stages in the sexual cycle of *Perna perna* at the Moroccan sites of Bouknadel, Mohammedia and Hay El Fath. Quantitative analyses were performed on the gonads, pedal ganglia and the cerebroid ganglia. The different stages (St0 + IIID; StI + II; StIIIA and egg-laying) correspond to an approximation of the % of mussels entered into the sexual stages based on the histological analysis obtained in another cohort collected at the same time (respectively July and September 2010 and March and April 2011). The data correspond to the mean ± sem of 8 independent measurements, each sample containing 5 individuals for each organ considered. Variation between sites; *p < 0.05, **p < 0.01, ***p < 0.001 is expressed with respect to the content measured from Bouknadel; +p < 0.05, ++p < 0.01, +++p < 0.001 to report difference between the content measured at Mohammadia and Hay El Fath (PLSD test).

We next describe the qualitative changes in monoamines across the reproductive cycle in these mussels from the site of Bouknadel (see Table 1 or Fig. 2). All monoamines and metabolites were present in the different organs of interest at all sexual stages (St0, StI + II, StIIIA and egg-laying). The three monoamines varied differently with sexual stage, and according to the organ and monoamine. In general, NA concentration was higher during gametogenesis and decreased sharply during egg-laying in the gonads and pedal ganglia, but not the cerebroid ganglia. Unlike NA, DA and 5-HT tissue concentrations were lower during the resting stage. These tissue levels began to increase during gametogenesis in the gonads and pedal ganglia until peaking during spawning, in particular in the cerebroid ganglia.

The DA metabolites 3,4-dihydroxyphenylacetic acid (DOPAC) and homovanillic acid (HVA) and the 5-HT metabolite 5-hydroxyindole-3-acetic acid (5-HIAA) were present in sample organs at the three sites. Table 1 reports levels in mussels from Bouknadel. Their levels were often very low, sometimes undetectable (in particular HVA), and displayed a higher variability compared to their parent compound. These levels also varied according to the tissue and showed seasonality (Table 1).

### Regional and seasonal fluctuations of monoamines in *Perna perna*

Figure 2 shows the monoamine content in gonads, pedal ganglia and cerebroid ganglia from the three distinct sites. In gonads, NA concentrations were always lower in Mohammedia and Hay El Fath mussels compared to Bouknadel mussels, whatever the maturation stage [F(2,23) = 54, 104, 18.5 and 8.6 for St0, StI + II, StIIIA and egg-laying; p < 0.001 for all stages except egg-laying where p < 0.01]. Indeed, NA concentrations were 3 to 10 times higher in mussels from Bouknadel before egg-laying. Despite the lower levels, the concentration of NA peaked during gametogenesis (about 300 to 400% of values measured in resting stage) at different stages in Mohammedia (StI + II; p < 0.001 compared to Hay El Fath) or Hay El Fath (StIIIA). Conversely, DA concentrations were significantly higher in mussels collected at the Mohammedia and Hay El Fath sites compared to those from Bouknadel during the resting stage [F(2,23) = 123, p < 0.001] and StI + II [F(2,23) = 18.5, p < 0.001], but not StIIIA [F(2,23) = 2.3, n.s.] and egg-laying [F(2,23) = 1.6, n.s.]. 5-HT concentrations were higher in Mohammedia and Hay El Fath mussels compared to Bouknadel mussels during the resting stage [F(2,23) = 30.7, p < 0.001], StI + II [F(2,22) = 8.9, p < 0.01] and egg-laying [F(2,23) = 3.8, p < 0.05], but not StIIIA [F(2,23) = 2.1, n.s.]. Like DA, 5-HT concentrations were extremely low in Bouknadel mussels (p < 0.001 compared to the other two sites at resting stage; PLSD test) and sharply increased during gametogenesis and egg-laying (10-fold increase). The concentrations of 5-HT in mussels from Mohammedia and Hay El Fath were between 50 and 100 pg/mg and were lower than those found in Bouknadel mussels (p < 0.05, PLSD test).

In the pedal ganglia (Fig. 2), concentrations of NA varied between the sites at rest and also evolved differently. For example, NA tissue content was significantly higher in mussels from Bouknadel during resting [F(2,23) = 12.7, p < 0.001], StI + II [F(2,23) = 38.3, p < 0.001], and egg-laying [F(2,23) = 24.6, p < 0.001] compared to mussels from Mohammedia and Hay El Fath (p < 0.001, PLSD test). A difference was also noted for StIIIA [F(2,23) = 9.4, p < 0.001], but values in Hay El Fath mussels were lower than those measured in Bouknadel (p < 0.001, PLSD test) and Mohammedia (p < 0.01) mussels. Interestingly, the DA content in pedal ganglia was similar at resting [F(2,20) = 2.1, n.s.], StI + II [F(2,23) = 0.4, n.s.] and StIIIA [F(2,23) = 1.7, n.s.] at all sites. DA content then increased progressively during gametogenesis. Differences between sites were found during egg-laying [F(2,23) = 3.8, p < 0.05]. Indeed, DA contents increased to 400 pg/mg at the Bouknadel and Mohammedia sites while they stayed significantly lower at Hay El Fath (p < 0.01, PLSD test). The 5-HT content in pedal ganglia was higher in mussels from Bouknadel at resting [F(2,22) = 8.8, p < 0.01] and StI + II [F(2,23) = 10.5, p < 0.001] compared to Mohammedia and Hay El Fath. While the 5-HT levels were similar during StIIIA [F(2,23) = 2.3, n.s.], a significant difference was observed during egg-laying between the sites [F(2,23) = 5.4, p < 0.05]. The increase in 5-HT content was progressive, reaching its maximum during spawning, except in mussels from Mohammedia.

The monoamine content in cerebroid ganglia from the different sites is shown in Fig. 2. The NA content in the cerebroid ganglia varied between sites during the resting phase [F(2,22) = 31.8, p < 0.001], StI + II [F(2,21) = 16.8, p < 0.001] and StIIIA [F(2,21) = 19.4, p < 0.001] but not during egg-laying [F(2,21) = 2.4, n.s.]. The U-shaped time course found in Bouknadel contrasted with the progressive increase in NA content from resting to egg-laying stages in mussels from the two other sites.

DA contents displayed similar time courses at all sites but differed according to the stage considered [F(2,22) = 13.8, p < 001 for resting; F(2,23) = 15.2, p < 001 for StI + II; F(2,23) = 9.1, p < 0.001 for StIIIA and F(2,20) = 6.5, p < 0.01 for egg-laying] (Fig. 2).

Similarly, 5-HT contents displayed similar time courses at all sites although values differed with stage [F(2,22) = 22.7, p < 001 for resting; F(2,23) = 15.2, 17.5 and 3.8 for StI + II p < 001, for StIIIA p < 0.001 and egg-laying p < 0.05, respectively]. The profile was similar compared to that for DA where the values for DA were higher in mussels from the Mohammedia site compared to the other two sites (PLSD test).

### Qualitative analyses of the relationships between tissue monoamine content, season and location

In order to determine whether the perturbation of the monoaminergic systems in mussels could be related to external environmental stresses, we applied discriminant statistical analyses. Stepwise discriminant analyses were made for gonads and cerebroid tissues separately, for all three monoamines across time. No discriminant function was found in pedal tissues.

In the gonads, after eight steps of the analysis and introduction of 12 variables, two discriminant functions (roots) were obtained. The variables with the greatest discriminative power with respect to the three sites and their coefficient Wilk (lambda), indicative of their power, were: NA content during St0 + IIID (λ = 0.032), StI + II (λ = 0.035) and StIIIA (λ = 0.030) and 5-HT content during St0 (λ = 0.043). The discriminant function identified could predict the mussels’ location highly significantly (Wilks’ λ = 0.0085, P < 0.00001) and was 99% successful in classifying the mussels according to their location. The standardized canonical discriminant function coefficients are shown in Table 2. The statistical significance of roots used for interpretation was established on the basis of χ^2^ tests of subsequent roots: χ^2^ = 78.67, P < 0.0000001 (2 roots included) and the 2d root alone was also significant: Wilks’ λ = 0.61; χ^2^ = 8.06, P < 0.05, thus having additional discriminant power.

**Figure 3:**
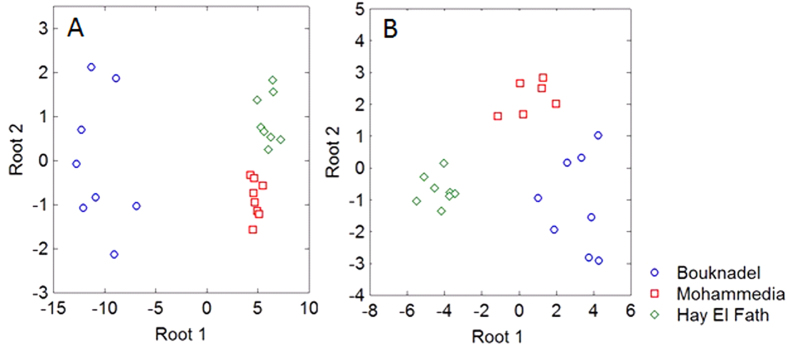
Scatter plots of the canonical values for root 1 vs root 2 for gonads (A) and cerebroid ganglia (B).

Based on the discriminant functions (roots) a scatter diagram of the canonical values for root 1 vs root 2 is shown in Fig. 3A. The elements with the greatest influence on root 1 in discriminating the mussels’ locations are NA and 5-HT contents during St0 + IIID, StI + II and StIIIA, whereas the second root discriminates between NA content during StI + II at the Mohammedia and Hay El Fath sites.

In the cerebroid ganglia, after eight successive steps of analysis and the introduction of 12 variables, two discriminant functions (roots) were obtained. The variables of the greatest discriminative power with respect to the three sites, as shown by the Wilk (lambda) coefficient were: 5-HT content during St0 + IIID (λ = 0.193) and StIIIA (λ = 0.332), DA content during StIIIA (λ = 0.412) and NA content during St0 + IIID (λ = 0.412). This discriminant function was 96% successful in classifying the mussels according to their location (Wilks’ λ = 0.025, P < 0.00001). The standardized canonical discriminant function coefficients are shown in Table 2. The statistical significance of roots used for interpretation was established on the basis of χ^2^ tests of subsequent roots: χ^2^ = 60.83, P < 0.0000001 (2 roots included) and the 2d root alone was also significant: Wilks’ λ = 0.30; χ^2^ = 19.84, P < 0.001, thus having additional discriminant power. The scatter diagram of the canonical values for root 1 vs root 2 is shown in Fig. 3B. The elements of the highest influence upon root 1 in discriminating the mussels’ locations were 5-HT and DA contents and also 5-HT content during St0, whereas the second discriminant root was represented principally by DA content during StIIIA and NA content during St0 + IIID.

As noted above, interactions between the cerebroid ganglia and gonads have been identified previously in the reproductive cycle. Their interaction could be indirectly revealed by studying the correlations between the tissue content of monoamine neurotransmitters in the same organ with other monoamines or in a different organ^15^. Indeed, the level of monoamines in a tissue cannot be interpreted directly^15^. Monoamines are made in monoaminergic cells under the influence of local factors and distal organs. It is suggested that the monoamine tissue content relates to a possible interrelationship with organs that should be low in resting conditions^15^. The seasonality of monoamine content reported here could highlight specific relations between organs.

**Figure 4:**
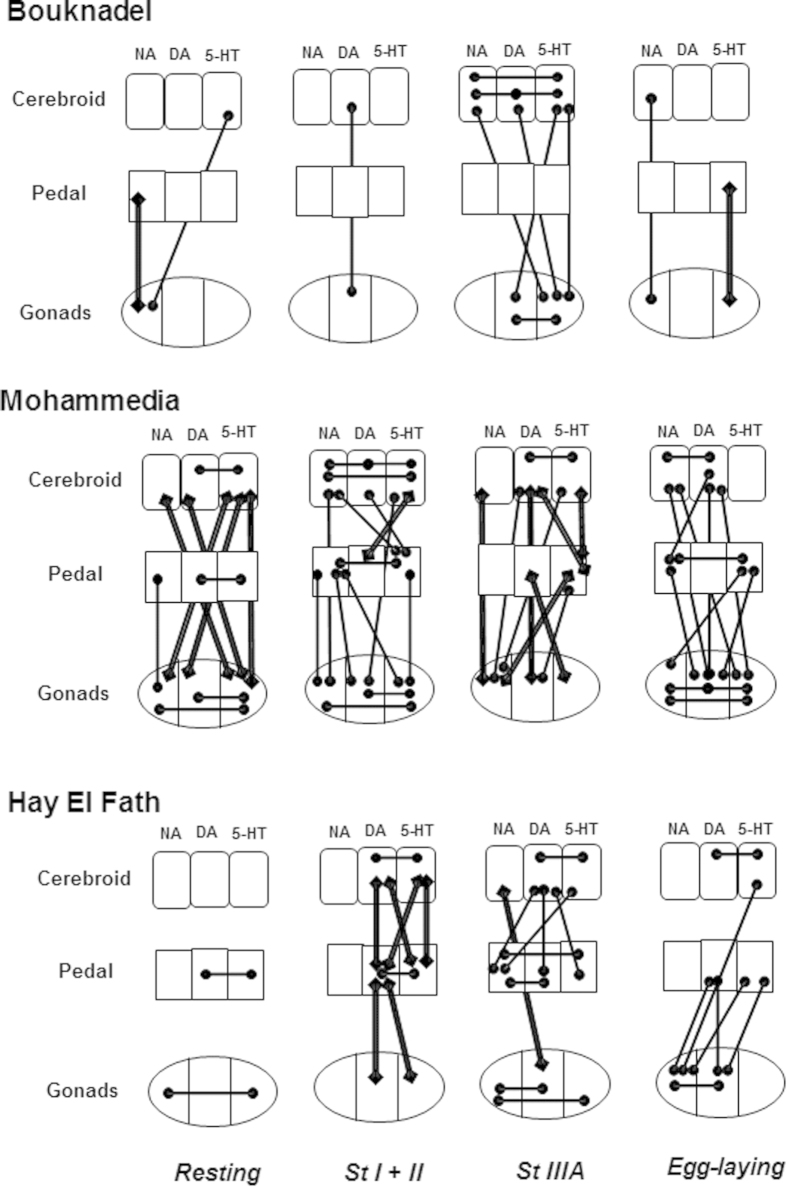
Correlation analysis of noradrenaline (NA), dopamine (DA) and serotonin (5-HT) content between organs at different stages of the sexual cycle of *Perna perna* at the Moroccan sites of Bouknadel, Mohammedia and Hay El Fath. The different stages (St 0 + IIID; StI + II; St IIIA and egg-laying) are approximate (see Fig. 2 legend). The Spearman’s rank order correlation test was used to establish correlations (p < 0.05) between individual measures. These correlations were either positive (plain line showing an association between cases) or negative (double lines).

Correlations for each stage were determined using the Spearman rank order test. Figure 4 illustrates the differences found between sites. At the Bouknadel site, very few correlations (13) between monoamine content were reported during the maturation process. Interestingly, relationships were found during StIIIA in cerebroid ganglia between all monoamines with respect to 5-HT and DA contents in gonads. Negative correlations have been reported solely between the pedal ganglia and gonads either at the resting stage with respect to NA content or after egg-laying with respect to 5-HT content. The most striking finding was the increase in the number of correlations between monoamine tissue content at Mohammedia (49) and Hay El Fath (27). The resting stage was marked by negative correlations of monoamine contents between cerebroid ganglia and gonads at the Mohammedia site and positive correlations between monoaminergic systems in the pedal ganglia or gonads at Hay El Fath. At these two sites, several correlations were also found between monoamines in the pedal ganglia.

## Discussion

In this study we provide neurochemical evidence that the monoamines DA, NA and 5-HT, which are present in the gonads, pedal ganglia and cerebroid ganglia of *Perna perna*, vary dramatically during the reproductive cycle. These variations are different for each of the three Moroccan sites examined and the differences could play a role in the changes in the reproductive cycles of mussels reported at these locations. Indeed, as well as quantitative changes, we have shown an influence of site on the temporal patterns of distribution of NA and 5-HT contents in the gonads and of all three monoamines in the cerebroid ganglia. We also found dramatic changes in the relationships between monoamines in the whole organism between sites. The increase in correlations at two sites could be due to stress and environmental constraints on the organisms, leading to disruption of the reproductive cycles.

Monoamine levels changed with the different stages of the sexual cycle (resting, developing, maturing and egg-laying) in the gonads and ganglia. Although the most abundant monoamine detected in *Perna perna* is DA followed by NA and 5-HT, we provide evidence that the timing of collection is an important factor. This seasonality might account for the variation in our results and those reported in the literature on bivalves^1^,^7^,^8^,^12^,^16^,^17^.

Bivalves like *Perna perna* may have three periods of spawning per annum and it is a characteristic of many bivalves to display variations in the timing of their reproductive cycles (supplementary Fig. S1). Thus, by combining samples from 5 individuals into a single analysis sample (n = 8 samples), such a grouped sample would be expected to represent the predominant reproductive state at the time of collection. This remains a source of variability because our interpretations are dependent on the distribution of individual stage differences within each cohort. Nonetheless, discriminant analysis revealed temporal and organ-specific changes in monoamine balance across the stages of the reproductive cycle, predictive of location-induced modifications. These discriminant parameters were identified in the gonads and the cerebroid ganglia, but not in the pedal ganglia, which normally does not play a role in bivalve reproduction. The temporal variations in monoamine concentrations found suggest that they follow, at least partially, the reproductive cycle, the stages of which are variably distributed over the year. One important finding of our study is that, at the Bouknadel site, there are fewer resting stages and more frequent egg-laying stages than at the Mohammedia and Hay El Fath sites. This is consistent with a previous study reporting that only two, rather than three, reproductive cycles were triggered at Mohammedia and Hay El Fath during 2007–2008 (unpublished data).

Marked variations in NA content were found in the gonads and pedal ganglia, with a 3 to 4-fold increase during gametogenesis and a drop during spawning. In agreement, increased NA has been measured in various tissues of the Pacific lion’s paw Scallop during gametogenesis stages (developing and maturing stages) and decreases at egg-laying^1^. Conversely, we found marked increases in 5-HT and DA contents in all tissues during advanced sexual stages and their levels remained elevated during spawning. 5-HT has been previously more closely associated with the later stages of gamete maturation and spawning^8^, while both DA and 5-HT are important during egg-laying and may induce spawning^5^,^18^,^19^,^20^. The increase in DA and 5-HT was found at similar levels in all tissues whereas the increase in NA in the gonads and pedal ganglia was in contrast to an inverse “U-shaped” profile found in the cerebroid ganglia. Although DA is the precursor for the synthesis of NA, the opposite variations of DA and NA occasionally detected in the three organs suggest that the measured contents of DA do not correspond to the precursor of NA. This hypothesis is further supported by the lack of systematic correlations between DA and NA content. Seasonality in DA and NA concentrations in gonads has been found in other bivalves^7^,^8^. The distinct temporal distributions of the three monoamines suggest distinct roles for all three monoamines in the process of reproduction^21^.

The seasonality of NA (gametogenesis and maturation) and 5-HT (maturation and spawning) found in mussels from Bouknadel is different from that found in mussels collected at the Mohammedia and Hay El Fath sites. Notably, NA concentrations were lower and 5-HT contents were less homogeneous over the course of the sexual cycle. Also, DA was lowest in gonads and 5-HT was highest in the cerebroid ganglia during egg-laying in mussels from Hay El Fath. The discriminant analysis showed that the monoamine content of the gonads and cerebroid tissue differed significantly between mussels according to their location, at specific time points in the reproductive cycle. In the gonads, NA concentrations before egg-laying and 5-HT content during the resting stage could differentiate between the sites from which the mussels originated. In contrast, in the cerebroid ganglia, 5-HT and DA contents during StIIIA were the discriminative variables along with NA content during St0 + IIID.

The existence of possible altered relationships between monoamines and organs with respect to location and seasons was confirmed using correlation analysis between individual samples^15^. At Bouknadel, very few correlations were found at the resting stage. Interestingly, correlations increased during late stages of maturation, suggesting that the systems cooperate with regard to 5-HT and DA gonad content before gamete release. Most of these correlations were positive and between monoamine content in gonads and cerebroid ganglia, consistent with their presumed role in the reproductive cycle. Again in contrast, the relationship between the pedal ganglia and gonad concentrations of NA and 5-HT during the resting and egg-laying stages was negative. The striking temporal relationship between monoamines in mussels from Bouknadel is absent in mussels from the other sites, with a dramatic increase in the number of correlations between monoamines and organs. It is also noticeable that relationships between monoamines are frequent in the pedal ganglia in Mohammedia and Hay El Fath. This finding suggests that at these sites, the pedal ganglia interfere with the activity of the cerebroid ganglia and gonads via monoaminergic signals that are used for purposes other than reproduction.

We postulate that the organisms are less able to enter reproductive activity when confronted by environmental constraints. Monoamines play essential roles in several physiological processes in molluscs and crustaceans, including feeding, locomotion, respiration, reproduction, inflammatory responses^1^,^11^ and even anxiety^22^. It is thus likely that monoaminergic signals not only differ between the sites of animal origin, but that pollution could be involved in qualitative and quantitative changes in mussel monoaminergic functions. Indeed, mussels in the region of Hay El Fath and Mohammedia are located near to domestic and industrial waste water outlets, respectively, and could suffer from anthropogenic stress. It has been reported that tissue levels of DA and 5-HT are decreased in the nervous system of bivalves exposed to heavy metals^11^,^23^. We have also reported that mean heavy metal concentrations in mussels over the course of a year are different for all sites, and generally higher at Mohammedia. Changes in organic solvents from domestic and municipal effluents could also account for the different environments. A direct alteration of enzymes or precursor elements is unlikely to be solely responsible for the reported differences because some tissue levels of monoamines are still elevated in mussels from both Mohammedia and Hay El Fath. Additionally, the marine environment might play a role at Mohammedia where salinity and conductivity were found to be lower compared to the other sites. Overall, a combination of several environmental factors probably accounts for the differences between sites^11^. Environmental conditions may therefore impact directly on the reproductive cycles via an alteration in the neuroendocrine function of monoamines, notably NA, as indicated by our analyses, or indirectly via an alteration in other, perhaps competing, biological functions modulated by monoamines.

## Conclusion

The present study provides important data regarding the function of monoamines in the reproductive cycle of *Perna perna* and how this can be disrupted depending on location. We are currently working at identifying other environmental factors involved in the alteration of monoamine function, whether related or not to industrial and domestic waste. Using qualitative and quantitative analyses, we provide strong evidence that monoamines display specific relationships between organ content and seasonality. Our data suggest that the more the monoamines are synchronized as a consequence of environmental changes, the less likely *Perna perna* is to enter a reproductive cycle. These descriptive and biochemical analyses could provide information about the quality of an environment for bivalves or other organisms, and lead to additional information regarding the suitability of seafood consumption for humans. Finally, the changes in monoamine patterns and relationships reported here also have fundamental implications for mammals regarding the influence of monoamines in various biological functions, including cognition^15^,^24^.

## Methods

### Animals

Studies were performed on 3 mussel populations of Moroccan *Perna perna* collected along the Atlantic coast of Morocco between Rabat and Casablanca. The first population was obtained at the site of Bouknadel, located 15 km north of Rabat. The second mussel bed was located at Mohammedia at the level of Oued El Maleh, a river 50 km south of Rabat. This site receives industrial wastewater. The third population was obtained from Hay El Fath on the Rabat coast. This site receives domestic wastewater. Mussels from Bouknadel were taken as a control because they were developing at a site that is, supposedly, not associated with a daily exposure to wastewater. A total of 40 adult mussels *Perna perna* (sexually mature, sizes: 40–60 mm) were collected in three different sexual stages: before, during and after egg-laying (July 2010, September 2010, March 2011 and April 2011). The timings of collection were selected according to the sexual stages (sexual cycle 2010–2011; see supplementary Fig. S1) of each population in order to evaluate the seasonal variation of monoamines before, during and after egg-laying (Fig. 1A).

### Chemicals

All monoamines and metabolites for standards for high pressure liquid chromatography with electrochemical detection (HPLC-ED) were purchased from Sigma (Sigma Chemical Co.,) or Fisher (Fisher Chemical Co.,).

### Determination of the stage of the reproductive cycle

Thirty dissected gonads per site were dipped into Bouin’s fixative (picric acid in distilled water 75%, formaldehyde 20% and acetic acid 5%) for 24 h and were dehydrated through a gradual series of ethanol and butanol baths. The pieces were then dipped in cytoparaffin. Seven μm-thick sections were cut using a microtome (micro Tec cut 4060) mounted on gelatin-coated slides, and stained using Pregnant-Gabe’s trichrome^25^. They were examined under a light microscope to determine the sexual stages according to the classification of Lubet^26^.

### Dissection and organs sampling

After the collection of mussels at the different sites and times, the samples were transported to the laboratory in bags and placed in a freezer at −20 °C. Gonads, pedal and cerebral ganglia were dissected and kept at −80 °C. Samples, placed in organs tissue tubes, were packed in a sealed polystyrene box containing dry ice (−80 °C). They were transported from Rabat to Bordeaux (University of Bordeaux) in order to measure their monoaminergic content.

### Neurochemical assessment of monoamine content

Tissue concentrations of the biogenic amines NA, 5-HT, DA with DA metabolites DOPAC and HVA and the 5-HT metabolite 5-HIAA were measured by HPLC-ED in the three distinct organs sampled. 5 mussels per pool (n pool = 8) were separated rapidly in a frozen chamber (−25 °C cryostat) the day of analysis. Organ tissues were thawed on ice, pulse sonicated for approximately 5 s in 400 μl of 0.1 M perchloric acid and centrifuged for 30 min at 13,000 rpm at 4 °C. Supernatants were diluted in the mobile phase (1/2 to 1/4 depending on the organ) and injected into the HPLC-ED system. The HPLC-ED procedure was performed as previously reported, with some modifications^27^,^28^.

Samples were kept at 4 °C on ice. They were injected (20 μl) into the HPLC column (Chromasyl C8, 150 × 4.6 mm, 5 μm; C.I.L.-Cluzeau, Sainte-Foy-La-Grande, France) protected by a Brownlee–Newgard precolumn (RP-8, 15 × 3.2 mm, 7 μm; C.I.L.-Cluzeau) using a manual injector (Rheodyne, model 7225i, C.I.L. Cluzeau) fitted with a 100 μl sample loop. The mobile phase, delivered at 1.2 ml/min flow rate using a HPLC pump (model Gold 106, Beckman) was as follows (in mM): 60 NaH2PO4 , 0.1 disodium EDTA, and 2 octane sulfonic acid plus 7% methanol, adjusted to pH 3.9 with orthophosphoric acid and filtered through a 0.22 mm Millipore filter. Detection of compounds was performed with a coulometric electrochemical detector (Coulochem II, ESA) fitted with dual-electrode analytic cell (model 5011, ESA). The potential of the electrodes was set at 350 and −270 mV, and the gain ranged from 100 to 200 nA. Signals were acquired on-line (Spike 2 software; Cambridge Electronic Design Ltd.) by a computer connected to the CED 1401 interface (Cambridge Electronic Design Ltd., Cambridge, UK)^22^.

The time for a sample elution was 30 minutes and standard solutions containing all the compounds of interest at known concentrations (from 100 pg to 1 ng/20 μl) have been regularly injected into the system (R value was greater than 99% for standards curves). A standard solution was injected after a series of 5 samples to verify the retention time of pics and the response. Series of samples for each organ, irrespective of their location of origin, were injected into the HPLC system. Samples were injected in duplicate. Under these conditions, the sensitivity for NA, DA, 5-HT, DOPAC, HVA and 5-HIAA was 5, 2, 15, 12, 35 and 10 pg/20 μl, respectively, with a signal/noise ratio of 3:1. Results were expressed in pg/mg of wet tissue.

### Statistical analyses

Data for the sexual stage of mussels at each site were expressed as a percentage of mussels at a specific sexual stage during a 13-month period of monitoring. The data combined from each time point were plotted using Tukey’s box with median, 25th and 75th percentiles, to show the inter-individual variability at each sexual stage. Differences in the proportion of mussels at a specific sexual stage between sites were analyzed using a Kruskall-Wallis analysis followed, in case of significance, by the Student-Newman-Keuls test. Data for each monoamine or metabolites in each organ tissue were expressed as the mean ± standard error of the mean (S.E.M.) for each sexual stage (or month). Descriptive analysis was done on monoamine content at the unpolluted sites in order to highlight fluctuations of monoamine content in sexual stages. A site effect on monoamine tissue content was analyzed using a one-way ANOVA for each time point relative to the sexual stage of mussels, considering the site as the main factor. In case of significant variation, the one-way ANOVA was followed by multiple comparisons using the Protected Least Significant Difference test (PLSD test). All differences were deemed significant at p < 0.05. An additional exploratory discriminant analysis^29^ was conducted to evaluate which of the 36 variables from the three monoamines in each tissue (gonads, pedal and cerebroid) across time, provided the best discrimination between the three locations. The discriminant analysis was performed using STATISTICA 7.1 software. The stepwise method was applied as follows. The established set of variables used for predicting the site of production (the model), was formed by deleting subsequent variables that contributed less to group discrimination. After keeping sufficient variables in the model (i.e. after obtaining maximum probability of a priori classification), discriminant functions (roots) discriminating mussel locations were calculated. The quality of the discriminant function was evaluated by Wilks’ lambda parameter, which is a multivariate analysis of variance statistics that tests the quality of group means for the variables in the discriminant function. The Wilks’ lambda parameter can assume values in the range of 0 (perfect discrimination) to 1 (no discrimination). The statistical significance of roots (discriminant functions) used for interpretation was established on the basis of χ^2^ tests of subsequent roots. Using statistically significant discriminant functions as the basis, canonical values were determined for discriminative variables. The scatter diagram of canonical values of subsequent cases for the two first roots determined in the course of the analysis was drawn to evaluate discriminant power of the model used.

Correlations, performed using the Spearman’s rank order correlation test, were made on the content of each monoamine. Correlations were considered significant at the 5% level.

## Additional Information

**How to cite this article**: Klouche, M. S. *et al*. Monoamine content during the reproductivte cycle of *Perna perna* depends on site of origin on the Atlantic Coast of Morocco. *Sci. Rep*. **5**, 13715; doi: 10.1038/srep13715 (2015).

## Supplementary Material

Supplementary Information

## Figures and Tables

**Table 1:** Monoamine concentrations in the gonads, pedal and cerebroid ganglia of mussels collected from the Bouknadel site.

Physiological variation of monoamines with respect to the reproductive cycles in the gonads and pedal or cerebroid ganglia of mussels at the Boukanadel site.				
	July (Resting)	September (St I + II)	March (St IIIA)	April (egg-laying)
NA gonads	128 ± 10.2	457 ± 34.2	304 ± 49	62.5 ± 4.5
NA pedal g	118 ± 31	227 ± 30	246 ± 44.5	70 ± 6.6
NA cerebroid g	90.7 ± 12	73 ± 9	55 ± 2.8	116 ± 13.6
DA gonads	15 ± 1	64 ± 3.4	241 ± 81	62.5 ± 4.5
DA pedal g	65.5 ± 11.9	125 ± 15.2	227 ± 54	450 ± 98.8
DA cerebroid g	158 ± 12.7	227 ± 19	132 ± 10.6	730 ± 46
DOPAC gonads	82 ± 11	157 ± 23	76 ± 19	64 ± 17
DOPAC pedal g	30 ± 5.6	59 ± 15	122 ± 55	18 ± 2.5
DOPAC cerebroid g	68 ± 14	28 ± 6.7	30.7 ± 6.6	122 ± 60
HVA gonads	77 ± 40	49 ± 25	51 ± 22	37 ± 12
HVA pedal g	14 ± 3.9	9 ± 4.6	7.8 ± 2.5	5.3 ± 2.7
HVA cerebroid g	8.3 ± 3.6	46.4 ± 13.6	13.2 ± 4	17.5 ± 4.2
5-HT gonads	8.7 ± 0.6	31 ± 5.7	142 ± 49.6	142 ± 14.3
5-HT pedal g	74 ± 16	115 ± 19.9	293 ± 54	302 ± 47
5-HT cerebroid g	51 ± 7	61 ± 6.5	63 ± 7	150 ± 9
5-HIAA gonads	188 ± 36	443 ± 70	29 ± 6	51 ± 5
5-HIAA pedal g	79 ± 22	122 ± 30	166 ± 46	56 ± 15.8
5-HIAA cerebroid g	56 ± 30	11.2 ± 1.4	46.5 ± 12	83 ± 39.5

Concentrations of NA, DA, 5-HT and the metabolites DOPAC, HVA and 5-HIAA were measured by HPLC assays and correspond to eight independent measurements for each period of the year. Each measurement was made on 5 mussels. The results correspond to the mean ± sem of the eight independent measurements.

**Table 2:** The standardized canonical discriminant function coefficients for the stepwise linear discriminant analysis for gonads and cerebroid ganglia.

	Root 1	Root 2
Gonads		
NA St0 + IIID	−1.07	0.68
NA StI + II	−0.87	−0.71
NA StIIIA	−1.18	0.38
5-HT St0 + IIID	1.02	−0.02
Cerebroid ganglia		
NA St0 + IIID	0.45	−0.76
5-HT St0 + IIID	−1.32	−0.75
5-HT StIIIA	−1.80	−0.67
DA StIIIA	1.66	1.15
