# Supplementary material for: Monoamine content during the reproductive cycle of Perna perna depends on site of origin on the Atlantic Coast of Morocco
Source: Sci Rep. 2015 Sep 9;5:13715. doi: 10.1038/srep13715 (PMC4563368; doi:10.1038/srep13715)
Supplement: Supplementary Information [file srep13715-s1.pdf]

Supplementary file (Klouche et al.)

Monoamine content during the reproductive cycle of *Perna perna* depends on site of origin on the Atlantic Coast of Morocco

Klouche M.S[1*]., De Deurwaerdère P[2,3*#]., Dellu-Hagedorn F.[2,4] Lakhdar Ghazal N[1]., Benomar S[1#]

[1]*Unit of Research on Biological Rhythms, Neuroscience and Environment; Faculty of Sciences, Mohammed V University, Rabat, Morocco;* [2]*University of Bordeaux,* [3]*CNRS, Institut des Maladies Neurodégératives UMR 5293,* [4] *CNRS, Institut des Neurosciences Cognitives et Intégratives d'Aquitaine UMR 5287, F-33000 Bordeaux, France.*

* The authors have participated equally in the study

Running title: Monoamines and bivalve mollusc reproduction

#Corresponding authors: Philippe De Deurwaerdère and Soumaya Benomar

E-mail: deurwaer@u-bordeaux.fr; s_benomar@yahoo.fr

Phone: +33 557 574 619

Determination of the stage of the reproductive cycle

As reported in the text, the determination of the sexual stages has been determined on approximately 30 mussels collected in each site and each month from May 2010 to April 2011. The distribution of the mussels in a particular stage was obtained after fixation, labelling, light microscope and according to the classification of Lubet[26] (see main text).

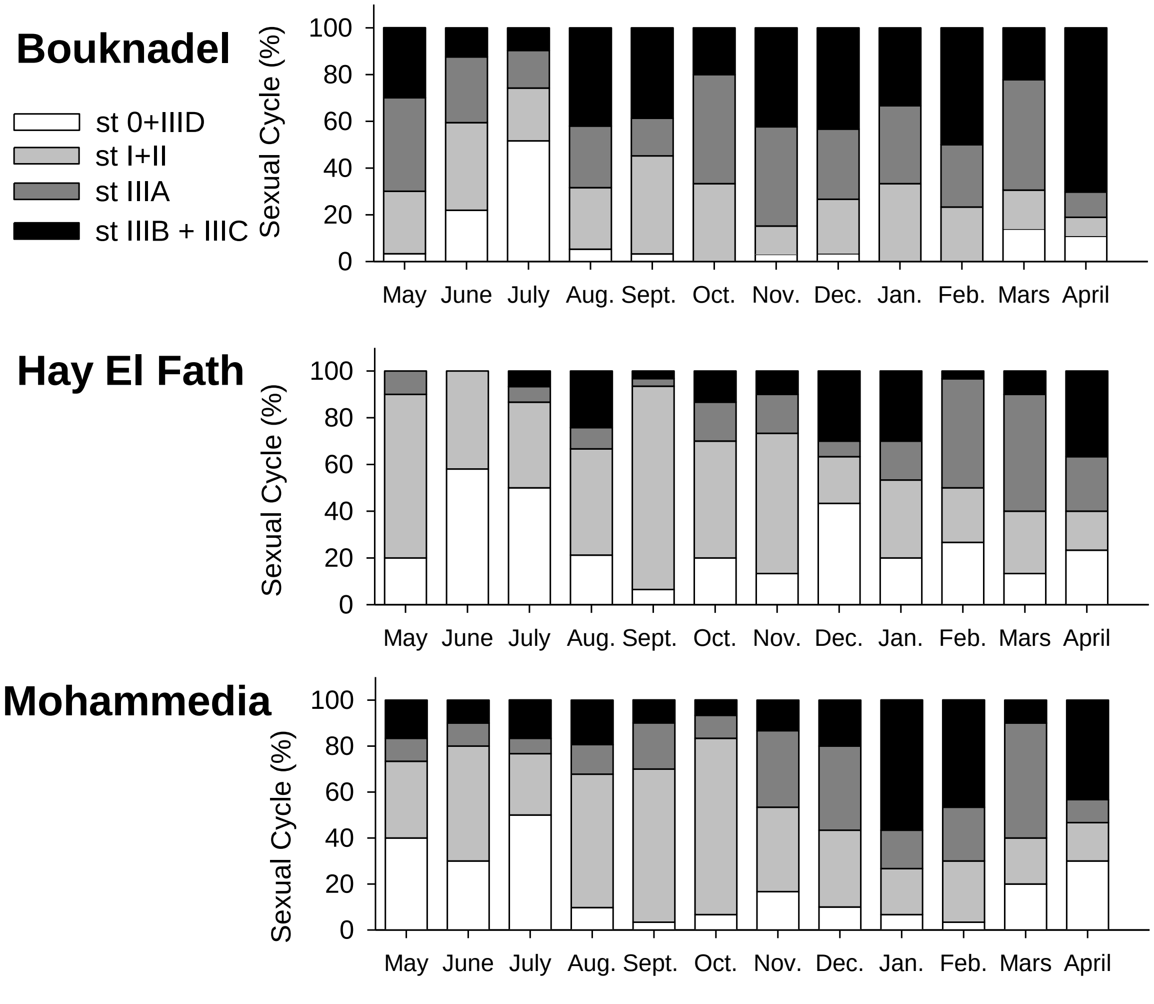

Supplementary Figure S1: Monthly variations of sexual stages noted in the gonads of *Perna perna* over a year in Bouknadel, Hay El Fath and Mohammedia. IIIC, gametogenesis redevelopment and spawning; IIIB, spawning; II + IIIA, later development + morphologically ripe; I, early development; 0 + IIID, resting stage + mussel recently spent. Black arrows indicate spawning periods at each site.

Environmental sea parameters and bioaccumulation of heavy metals

Salinity (°/00), conductivity (µS/cm), pH and sea temperature were acquired in situ during sampling in each site using ORION 3 STAR Portable Conductivity meter (accuracy: ± 0.5% ± 1 digit 0.01µS / cm). The conductivity values are then normalized, with the correlative coefficient (in Rodier, 1984) at 25 ° C.

For the bioaccumulation of heavy metals, samples were collected monthly in the 3 stations from April 2010 to April 2011. Each samples consisted of a pool of 50 mussels *Perna perna* 30-60 mm in size collected at low tide, transported to the laboratory in packed in ventilated seawater, in the laboratory mussels were cleaned, purged and stored in plastic bags at -20 C°. The soft tissues were removed from the shells in the laboratory, dried individually at 60 °C (at least for 48 h), pooled, ground and homogenized in a porcelain mortar. Metals analyses were performed at the Unit of Technical Support for Scientific Research in Rabat Morocco (UATRS). Aliquots of 200 mg of dried samples were mineralized at 90°C by nitric acid under atmospheric pressure. Metals were analyzed by atomic absorption spectrometry (Jobin Yvon Model: Ultima 2). Quantities were calculated from calibration curve using known concentrations of solutions same element. Quality assurance relies on the control of blanks and the accuracy and reproducibility of data relative to certified reference material.

Expression of the data and statistics

A single data has been collected for each month and for each site regarding sea factors and heavy metals. These data were combined from April 2010 to April 2011 (13 months). The variability of the 13 values in each site has been illustrated by Tukey's box with median, 25th and 75th percentiles. A significant difference regarding sea factors and heavy metals between sites was analysed using a Kruskall-Wallis analysis followed, in case of significance, by the Tukey's test for multiple comparisons (Supplementary Figures S2 and S3).

Results

The supplementary Fig. S2 reports that the values of sea temperature and pH were similar at all three sites with a median around 20.7 °C ($H=0.058$, ns) and 7.7 ($H=2.29$, ns), respectively. A drop in pH values was noticed at Mohammedia and Hay El Fath in March 2011 without impacting the overall profile over the year. In contrast, salinity ($H=18.8$, $p<0.001$) and conductivity ($H=13.65$, $p=0.001$) were significantly lower at Mohammedia compared to Bouknadel and Hay El Fath. This is noticeable during the months that have been selected to collect mussels for neurochemical analyses.

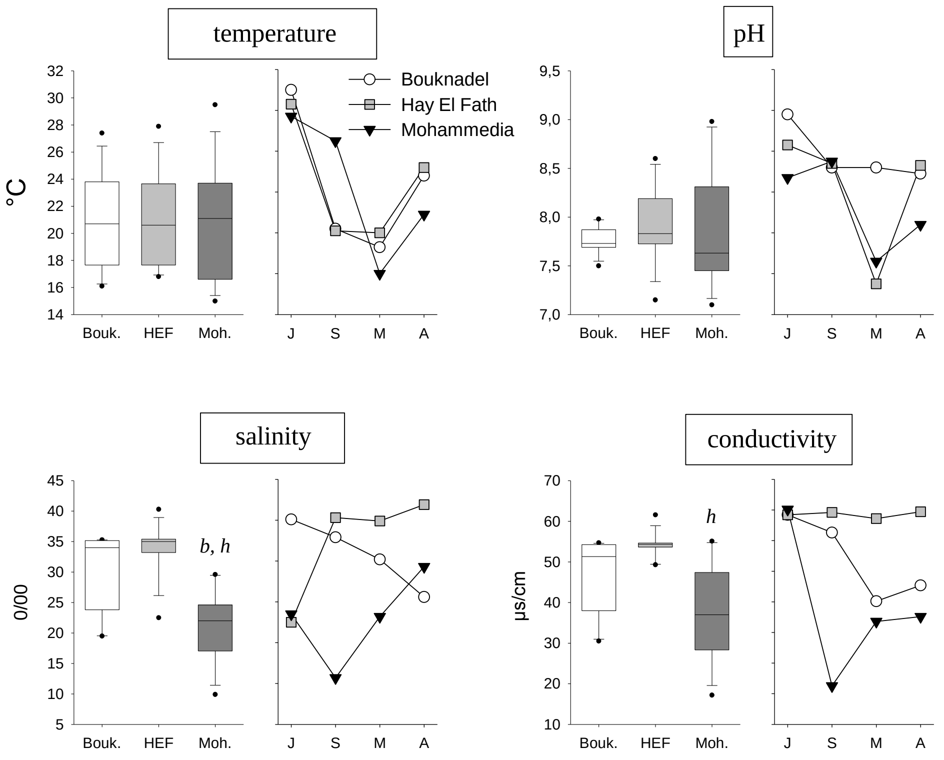

Supplementary Figure S2: Values of physico-chemical parameters (temperature, pH, salinity and conductivity of water) over a year (left graphs) or during the months used for the neurochemical study [right graphs; July (J) and September (S) 2010 and March (M) and April (A) 2011) at the sites of Bouknadel (Bouk.), Hay El Fath (HEF) and Mohammedia (Moh.). The data correspond to the median values recorded for a year or each month in case of the monthly values. The monthly variability in physico-chemical parameters for each site is illustrated by Tukey’s boxes. The symbols (h, m and b; $p<0.05$) refer to the site for which there is a statistical difference (Tukey’s test after significant Kruskall-Wallis Analysis).

The supplementary Fig. S3 reports the concentrations of heavy metals at the different sites over a year. In general, the monthly values were variable from month to month (see figure corresponding to the values recorded in the months selected for neurochemistry). The study of the overall concentrations for a year indicated that for all heavy metals except mercury ($H= 0.58$, ns) the values were different between sites including Zn ($H= 16$, $p<0.001$), Ni ($H= 16.7$, $p<0.001$), Pb ($H= 12$, $p<0.01$), Fe ($H= 7$, $p<0.05$), Mn ($H= 8.7$, $p<0.05$), Cr ($H= 25$, $p<0.001$), Cu ($H= 9.5$, $p<0.01$) and Cd ($H= 11.9$, $p<0.01$). The Pb concentrations were higher at both Mohammedia and Hay El Fath. Otherwise, the pattern of concentrations was distinct for each heavy metal.

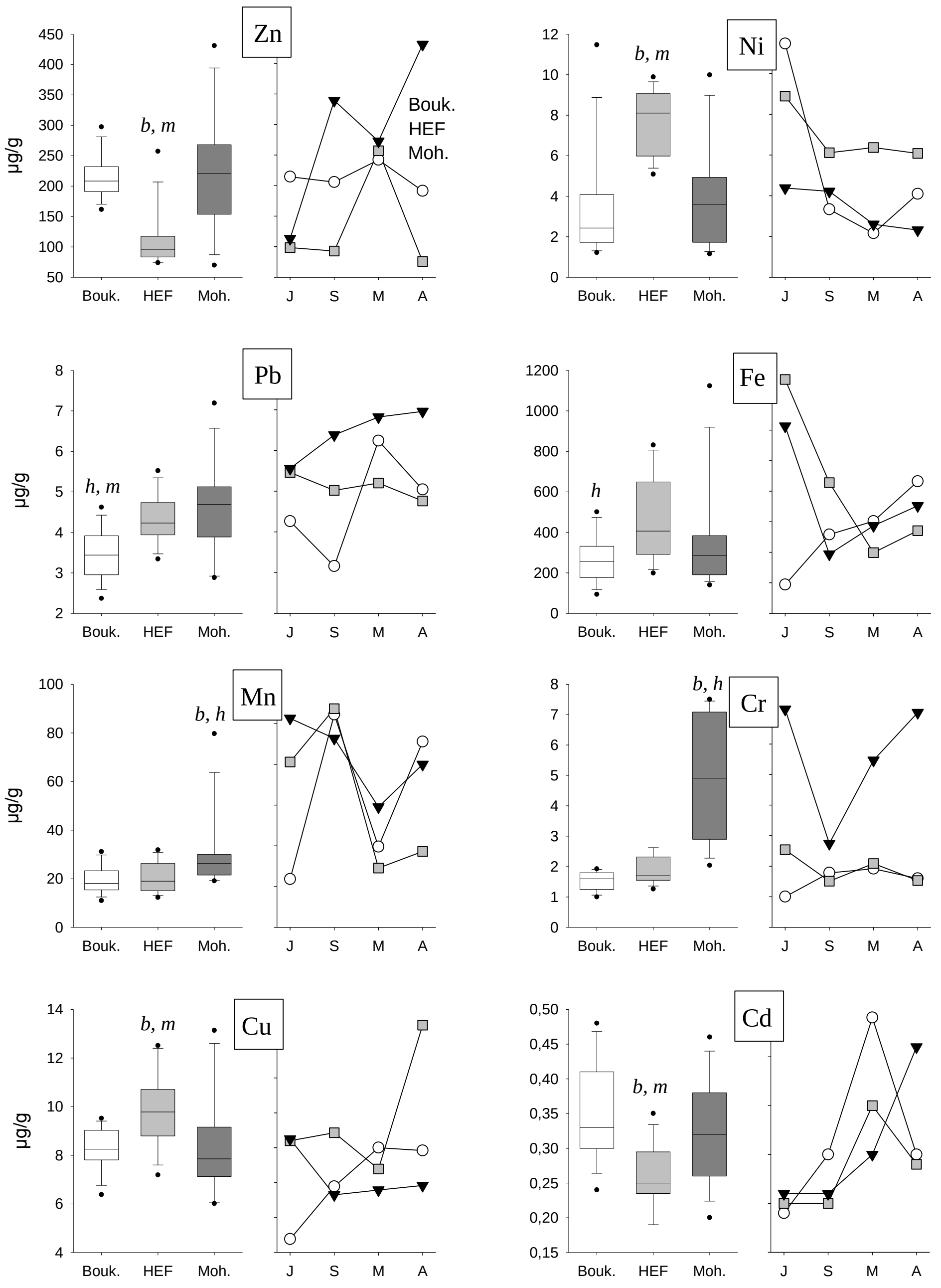

Supplementary Figure S3: Metals concentrations in the mussel P. perna of Zinc (Zn), Nickel (Ni), Lead (Pb), Iron (Fe), Manganese (Mn), Chrome (Cr), Copper (Cu), and Cadmium over a year (left graphs) or during the months selected for the neurochemical study [right graphs; July (J) and September (S) 2010 and March (M) and April (A) 2011) in the sites of Bouknadel (Bouk.), Hay El Fath (HEF) and Mohammedia (Moh.). The data, in µg.g^{-1} dry weight sample containing 50 mussels, correspond to the measurements of each element by atomic absorption spectrophotometry (AAS). The monthly values obtained for a year of monitoring have been combined for each element and for each site. The inter-month variability in metals concentrations is illustrated by Tukey's boxes for each site. The symbols (h, m and b) represent statistical differences between sites ($p<0.05$). The symbols (h, m and b; $p<0.05$) refer to the site for which there is a statistical difference (Tukey's test after significant Kruskall-Wallis Analysis).
